# A paired survey study on community perceptions of clinical trials: Shaping outcomes across medical fields

**DOI:** 10.1017/cts.2025.10199

**Published:** 2025-11-12

**Authors:** Mallory Von Lotten, Meredith Burns, Passiah L. White, Tiffany Mayo

**Affiliations:** 1 Heersink School of Medicine, University of Alabama at Birmingham, Birmingham, AL, USA; 2 Department of Dermatology, University of Alabama at Birmingham, Birmingham, AL, USA

**Keywords:** Clinical trials, health equity, skin of color, community education, minority participation

## Abstract

Underrepresentation of people of color in clinical trials limits equity in research and treatment outcomes. This study evaluated the impact of a brief, community-focused educational intervention on perceptions and willingness to participate. Participants attended 30-minute sessions (9 virtual, 2 in-person). Identical pre- and post-surveys were analyzed using paired t-tests with Bonferroni correction. Eighty-three participants (90.5% Black, 88.4% female; mean age 46.3) showed significant improvements in comfort with participation, randomization, belief in protections, willingness to participate, and comfort in skin-related trials (all *p* < 0.05). Brief education may improve understanding and participation attitudes in underrepresented groups.

## Introduction

People of color are significantly underrepresented in clinical trials. In the United States, Black participants comprise approximately 5% of all clinical trial enrollees, despite representing over 13% of the population [1]. This lack of diversity not only limits the generalizability of research findings but also reinforces disparities in treatment efficacy, safety data, and access to cutting-edge therapies.

The roots of clinical trial hesitancy are complex and multifactorial. Historical abuses, such as the Tuskegee Syphilis Study and the case of Henrietta Lacks, coupled with ongoing experiences of structural racism and healthcare inequities have contributed to a pervasive mistrust of the medical and research communities. In addition, many individuals report limited knowledge of clinical trial opportunities, confusion about participant rights and protections, and minimal communication from healthcare providers about research participation [2].

Educational outreach represents a promising, scalable strategy to address clinical trial hesitancy in underserved communities. A collaborative study by Clark LT et al has shown that culturally tailored, community-engaged interventions can improve understanding and shift attitudes about research participation [2]. However, few studies have specifically examined how brief educational sessions delivered by physician investigators affect perceptions of clinical trials in real-world community settings.

This study aimed to assess baseline perceptions [2] of clinical trials and evaluate the impact of an educational intervention among predominantly people of color. By engaging participants in active discussion, we sought to improve understanding, build trust, and increase self-reported willingness to participate in clinical research.

## Materials and methods

This cross-sectional study was approved as IRB-exempt by the University of Alabama at Birmingham Institutional Review Board (IRB-300009306). We assessed the impact of a brief educational intervention on perceptions of clinical trials among community members, primarily individuals identifying as people of color.

### Participants and setting

Between September 2023 and May 2025, a total of 11 educational sessions were conducted – 9 virtually via Zoom and 2 in-person at local community centers. Participants were recruited through a combination of grassroots outreach and established community partnerships with local nonprofits, student groups, and faith-based organizations across Alabama and surrounding regions. Any participant who identified as a person of color of any education level was included in the study and was offered a $10 incentive upon completion. Survey results from those who did not complete both pre- and post-surveys were excluded.

### Intervention

The intervention consisted of a 30-minute educational session developed by study team members. Presentations were preceded by an 11-item questionnaire and delivered by a physician investigator with occasional co-facilitation by a medical student presenter. The presentation covered the importance of clinical trials, underrepresentation of skin of color populations in clinical research, review of historical injustices, discussion of ethical protections, explanation of clinical trial phases, evaluation of common misconceptions, and summary of potential risks and benefits of trial participation. Sessions concluded with an open question-and-answer session to address participant concerns and encourage discussion, along with a post-session survey identical to the pre-session survey.

### Survey instrument

A paired survey study utilized an identical 11-item Likert-scale questionnaire to assess participant perceptions pre- and post-intervention. Questions evaluated participants’ understanding of clinical trial processes, comfort with participation, trust in regulatory protections, and willingness to engage in trials. Responses were scored from 1 (“strongly disagree”) to 5 (“strongly agree”).

### Data collection and analysis

Surveys were distributed via Qualtrics, and responses were collected anonymously for analysis. Descriptive statistics and paired-sample t-tests were used to assess differences in pre- and post-intervention survey responses across 11 matched Likert-scale items. Each question was scored on a 5-point scale from “strongly disagree” (1) to “strongly agree” (5). Only participants who provided a response for both the pre- and post-intervention versions of a question were included in that item’s analysis. Paired comparisons were conducted independently for each question. A *p*-value of <0.05 was considered statistically significant. All statistical analyses were performed using Python (v3.11) and the SciPy statistical library.

## Results

A total of 98 individuals completed the full pre- and post-intervention survey. Of these, 95 provided complete demographic information. The mean participant age was 46.3 years (range: 18–81). The majority identified as female (88.4%) and Black or African American (90.5%). Most respondents reported some college education or higher with 61.1% holding a bachelor’s degree or above. Approximately half (48.4%) had previously participated in a clinical trial. Participant characteristics are summarized in Table 1.

**Table 1.** Demographic characteristics of total initial survey respondents (*N* = 95). Percentages are based on available responses

Characteristic	*N* or %
Age (mean ± SD)	46.3 ± —
Age range	18–81
Gender – Female	88.4%
Gender – Male	11.6%
Race – Black or African American	90.5%
Race – Multiracial	5.3%
Race – White	2.1%
Race – Native Hawaiian or Pacific Islander	1.1%
Ethnicity – Not Hispanic or Latino	98.9%
Ethnicity – Hispanic or Latino	1.1%
Education – Bachelor’s Degree	29.5%
Education – Master’s Degree	25.3%
Education – Doctorate	6.3%
Education – Some college	18.9%
Education – Associate Degree	9.5%
Education – High school diploma or GED	7.4%
Education – Less than high school diploma	3.2%
Prior Clinical Trial Participation – Yes	48.4%
Prior Clinical Trial Participation – No	51.6%

After data cleaning, 83 participants were included in the final paired *t*-test analysis for each of the 11 matched Likert-scale items. Five items demonstrated statistically significant improvement following the intervention:

- Comfort participating based on current knowledge (Mean: 3.48 → 3.78, *p* = 0.008)
- Comfort with trials involving randomization (Mean: 2.86 → 3.19, *p* = 0.0079)
- Belief that rules and regulations protect participants (Mean: 3.67 → 3.92, *p* = 0.0263)
- Willingness to participate in future trials (Mean: 4.28 → 4.40, *p* = 0.0322)
- Comfort participating in skin-related trials (Mean: 3.66 → 4.07, *p* < 0.0001)

**Figure 1.**
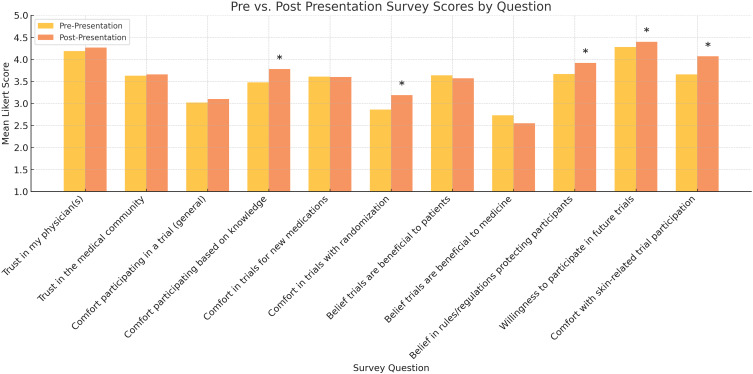
Visualization of pre- and post-presentation Likert-scale responses across 11 matched survey items, illustrating changes in perceptions following the educational session. Asterisks (*) indicate statistically significant differences (*p* < 0.05).

The remaining items did not show statistically significant differences, though most trended positively. Full results for all survey items are presented in Table 2 and Figure 1.

**Table 2.** Pre- and post-intervention survey responses across 11 matched Likert-scale items (*N* = 83). Scores range from 1 (“strongly disagree”) to 5 (“strongly agree”). Asterisks (*) indicate statistically significant improvements (*p* < 0.05)

Survey question	Pre (mean ± SD)	Post (mean ± SD)	Mean Δ	p-value	Significant
I trust my physician(s).	4.19 ± 0.74	4.27 ± 0.45	0.08	0.3212	
I trust the medical community at large.	3.63 ± 0.74	3.66 ± 0.75	0.04	0.6575	
I feel comfortable participating in a clinical trial in general.	3.02 ± 1.21	3.1 ± 1.04	0.07	0.3689	
I feel comfortable participating in a clinical trial based on my current knowledge.	3.48 ± 0.87	3.78 ± 0.92	0.3	0.008	*
I feel comfortable participating in a clinical trial involving new medications.	3.61 ± 1.11	3.6 ± 1.16	−0.01	0.8706	
I feel comfortable participating in a clinical trial involving randomization.	2.86 ± 0.94	3.19 ± 1.04	0.34	0.0079	*
I believe clinical trials are beneficial to patients.	3.64 ± 1.01	3.57 ± 1.10	−0.07	0.5838	
I believe clinical trials are beneficial to the field of medicine.	2.73 ± 0.99	2.55 ± 0.95	−0.18	0.1285	
I believe there are rules and regulations in place to protect clinical trial participants.	3.67 ± 0.81	3.92 ± 0.91	0.24	0.0263	*
I would be willing to participate in a clinical trial in the future.	4.28 ± 0.77	4.4 ± 0.76	0.12	0.0322	*
I would feel comfortable participating in a clinical trial related to skin conditions.	3.66 ± 0.86	4.07 ± 0.79	0.41	0	*

## Discussion

This study demonstrated that a brief, community-focused educational intervention has the potential to shift perceptions about clinical trial participation among individuals from historically underrepresented groups in select areas. Of the eleven matched pre- and post-survey items, five demonstrated statistically significant improvement in the following items: participating based on current knowledge, comfort with randomization, belief in participant protections, willingness to enroll in future trials, and comfort participating in both general and dermatology-specific research. These areas reflect some of the most commonly cited barriers to trial participation and suggest that targeted education may serve as a key lever for change. All areas, with the exception of participation in clinical trials related to skin conditions, reject the null hypothesis with p-values exceeding the Bonferroni correction value of 0.00455.

These findings reinforce the importance of addressing clinical trial hesitancy not solely through recruitment strategies but also through education. Mistrust in clinical research is often rooted in historical injustice and compounded by ongoing disparities in care. Thus, trust must be intentionally cultivated. Educational interventions that frame healthcare providers and trainees as community partners, rather than institutional authorities, may be uniquely effective in dismantling this mistrust. In addition, survey results demonstrated a high level of physician trust, emphasizing the role of physicians in improving patients’ perceptions and awareness of clinical trials.

The intervention’s structure – delivered by a skin of color physician investigator with occasional support from a medical student – may have further bridged generational and cultural gaps. Its brevity and adaptability allowed for replication across multiple community settings, both virtual and in-person, enhancing its scalability and relevance in diverse contexts.

Nonetheless, several limitations must be acknowledged. The sample size, while respectable, remains modest. Furthermore, the predominance of female participants and monetary incentive may have introduced selection bias and limited generalizability of the study. Future studies should incorporate larger sample sizes with a comparable number of male and female participants and investigate whether positive perceptual shifts translate into actual clinical trial enrollment. Additionally, future studies should incorporate controls to isolate intervention impact, and incorporation of a long-term follow-up or control group to assess sustained behavioral impact or isolate causality.

Ultimately, increasing representation in clinical trials is not only a scientific imperative – it is a moral one. This study offers a replicable, community-driven model that can be adapted across specialties and populations to foster trust, expand access, and promote equity in research.

## Conclusion

A brief community-based intervention on clinical trials addressing historical injustices, common misconceptions, and legal protections was shown to improve understanding of and attitudes toward clinical trial participation in a minority population. Survey results demonstrated a moderate level of physician trust, emphasizing the role of physicians in improving clinical trial awareness and participation. These findings support the utility of educational outreach to address disparities in research representation.
